# Determinants of uterine rupture among cases of Adama city public and private hospitals, Oromia, Ethiopia: a case control study

**DOI:** 10.1186/s12978-018-0606-4

**Published:** 2018-09-27

**Authors:** Fikru Abebe, Ephrem Mannekulih, Abebe Megerso, Abdurahman Idris, Tsegaye Legese

**Affiliations:** 1 Department of Gynecology and Obstetrics, Adama Hospital Medical College, Adama, Ethiopia; 2 Departments of Public Health, Adama Hospital Medical College, Adama, Ethiopia

**Keywords:** Adama, Hospital, Uterine rupture

## Abstract

**Background:**

Ethiopia is among the ten world countries with highest maternal death rates that accounts for more than 59% of global maternal deaths. Uterine rupture is one of the dangerous obstetric problems with high potential of causing maternal and neonatal morbidity and mortality. The case fatality rate of uterine rupture is high and hence identifying factors associated with uterine rupture remains important to guide decision makers and practitioners. The study aimed to identify factors associated with uterine rupture among clients managed in Adama city public and private hospitals during January 2011 to December, 2015.

**Methods:**

Unmatched case control study design was employed. The sample size was determined using computer software considering the basic statistical assumptions and accordingly a total of 432 women, (144 with uterine rupture as cases and 288 with spontaneous vaginal delivery as controls) managed in all hospitals during the study period were included in the study. A data collection tool that contains available variables was designed and used to extract data from log books and client cards. Data were entered into EPI-Info-7 and exported to Stata-12 for cleaning and analysis. The study participants were characterized using descriptive statistics. The associations between uterine rupture and independent variables were modeled using binary logistic regression analysis. The association between independent variables and uterine rupture was estimated using odds ratio with 95% confidence intervals. The statistical significance of the association was declared at *P*-value < 0.05.

**Results:**

The odds of having a uterine rupture were found to be more than six times higher among rural residents (AOR = 6.29; 95% CI: 3.39, 11.66) compared to urban. Other independent predictors include gravidity of five or more (AOR = 27.89; 95% CI: 8.42, 92.34), having a history of cesarean section scar (AOR = 9.94; 95% CI: 3.39, 11.66) and not having an antenatal care visit (AOR = 9.64; 95% CI: 4.37, 21.29).

**Conclusion:**

Rural residence, multigravidas, cesarean section scar and not having an antenatal care visit were independent predictors of uterine rupture in the current study. Therefore, improving access and strengthening essential obstetric care, antenatal and family planning services with complete packages are crucial interventions in the reduction of the odds of having uterine rupture. In addition, the strengthening of the referral system is mandatory for women residing in rural areas.

## Plain english summary

### Background plain

Ethiopia is among the ten world countries with the highest number of maternal deaths that accounts for more than 59% of global maternal deaths. Uterine rupture is one of the dangerous obstetric problems with high potential of causing maternal and neonatal death. This study aimed to identify factors associated with uterine rupture among clients managed in Adama city public and private hospitals during January 2011 to December, 2015.

### Methods

The study was employed by comparing cases (women with uterine rupture) and controls (women with normal spontaneous vaginal delivery). A total of 432 women, (144 cases and 288 controls) managed in all hospitals during the study period were included in the study. Data were analyzed using STATA-12 computer soft. The characteristics of women participated in the study were descriptive using frequency distribution. A statistical analysis method called logistic regression was used to assess the associations between uterine rupture and independent variables. The chance to develop uterine rupture was estimated by odds ratio.

### Results

The higher chance of developing uterine rupture was observed among women from rural residents compared to urban, women with large number of pregnancies, women who have scarred uterus due to previously delivered by operation and women never attend antenatal care for the current pregnancy.

### Conclusion

Therefore, improving access and strengthening essential care during pregnancy and labor and family planning services with complete packages are crucial interventions. In addition, strengthening strong referral system is mandatory for women residing in rural areas.

## Background

Uterine rupture is defined as a full-thickness separation of the uterine wall and the overlying serosa [1]. It is a catastrophic obstetric complication associated with high rates of maternal morbidity and mortality. Ethiopia was the fourth among ten countries collectively accounted for 59% of all maternal deaths worldwide [2]. Uterine rupture was one of the top four causes that attributed to 36% of these maternal deaths in addition to hemorrhage (22%), hypertensive disorders of pregnancy (19%) and sepsis/infection (13%) [3, 4]. This unfortunate event has remained the most significant problem in developing nations [5].The overall incidence of uterine rupture is higher in developing countries than in developed countries, which is around 74 in 10,000 [6, 7]. Uterine rupture usually occurs during labor but it can also occur during pregnancy [8]. It has also been reported in all trimester of pregnancy [9]. Uterine rupture in primi-gravida with no identifiable risk factor has also been reported [9]. The signs and symptoms of uterine rupture depend on the timing, site, and extent of uterine defect. The classical signs and symptoms of uterine rupture include fetal distress, loss of uterine contraction, abdominal pain, hemorrhage, recession of the presenting fetal part and shock. The initial signs and symptoms are however, non-specific, a condition that makes diagnosis difficult, which sometimes delays definitive therapy. This delay in diagnosis and treatment often leads to adverse maternal and perinatal outcome. It is therefore important to maintain a high index of suspicion [10, 11].

Uterine rupture is one of the dangerous obstetric problems with higher potential of causing maternal and neonatal morbidity, if not death. Case fatality rate for uterine rupture is as high as 30.4% [12]. Consequences of uterine rupture depend on the time between diagnosis of uterine rupture and delivery. It has been postulated that from the time of diagnosis to delivery only 10–37 min are available before clinically significant fetal morbidity becomes inevitable due to catastrophic hemorrhage or fetal anoxia. Uterine rupture contributes significantly to maternal morbidity and mortality, and perinatal mortality. Fatal consequences are admitted to the neonatal intensive care unit, fetal hypoxia or anoxia, and neonatal death. Maternal consequences are hemorrhage, hypovolemic shock, bladder injury, need for hysterectomy, and maternal death. Morbidity and mortality following rupture of the uterus depend on level of medical care [13–16].

Studies reported that about 60% of uterine rupture can occur spontaneously and there were also 29% scar rupture and 11% traumatic rupture [9, 17]. Several studies associated the odds of having uterine rupture with maternal education [18, 19], increased in maternal age [6, 9], distance from health care facility [6, 9, 18, 19], multi-gravidity [6, 9, 20, 21], increased gestational age [6, 9], not utilizing antenatal care [6, 9, 21], high parity [4–6, 18, 19, 22], having previous cesarean section [4, 5, 17, 18, 22–24], mal-presentation [4–6, 18, 22], unsafe obstetric practices such as inappropriate use of oxytocins, prostaglandins drugs for induction/augmentation of labor and fundal pressure in prolonged second stage [6, 18, 20, 21]. Studies also relate congenital uterine anomalies, feto-pelvic disproportion, breech extraction, previous myomectomy, fetal macrosomia, neglected labor and uterine instrumentation with the odds of having uterine rupture [4–6, 18, 22].

Evidences on factors associated with uterine rupture are scarce in our study setting. Therefore, the results of this study will inform both clinical practitioners and maternal health program planners on important areas of attention, and hence, contribute to the reduction of maternal morbidity and mortality. Moreover the current study will also serve as one sample study for meta-analysis studies aiming to identify the common predictors of uterine rupture.

## Methods

### Study setting, period and design

Hospital based unmatched case control study design was used to assess factors associated with uterine rupture among mothers given birth and managed at Adama city, one public and four private hospitals during January 2011 to December, 2015. Adama city forms an especial zone of Oromia region and located 99 km south east of Addis Abeba (Capital city of Ethiopia). Based on 2007 census conducted by the Central Statistical Agency (CSA) of Ethiopia [25], this city has a total population of about quarter a million. The city has one governmental and three private hospitals. These hospitals together serve over five million catchment populations and serve as a referral site for neighboring zones and regions (Affar, Amhara and Somali). The hospitals have an operation room with nine functional operation room tables, nine gynecologists, 15 anesthetists and 30 midwives. Average annual numbers of deliveries of all type in these hospitals on average was 8, 320.

### Study participants

All mothers delivered in Adama city governmental and private hospitals during the study period were considered as a source population for the current study. Women with uterine rupture were taken as cases and those with a normal spontaneous vaginal delivery (registered following each uterine rupture case) were considered as controls. Sample size for unmatched case control study design was calculated using EPI Info version 7 [26], considering all the following assumptions;

- Power of the study = 80%,
- Confidence interval = 95%,
- Case – to- control ratio = 1 to 2,
- Percentage of controls exposed to risk factor (cesarean section scar in this case) = 35%,
- Odds ratio to be detected = 1.82 (planned detection capacity of our study),
- Percentage of cases exposed (has cesarean section scar in this case) = 49.55% (from relevant literature) [17].

Accordingly, a total sample size of 432 women that is 144 cases of uterine rupture and 288 normal spontaneous vaginal deliveries were included in the study. The sampling frame was developed based on the client’s medical registration number recorded on the log books in the labor wards and operating rooms over the 5 years from January 2011 to December, 2015. Using this frame, mothers diagnosed for uterine rupture and managed in the selected hospitals and the two subsequent mothers who had normal spontaneous vaginal delivery were selected as cases and controls respectively.

### Data collection

Data were extracted from both the log books and the client cards which had been recorded over the 5 years from January 2011 to December, 2015. As sources of data the medical records of the selected cases that were diagnosed for uterine rupture and the medical records of two subsequent mothers with spontaneous vaginal delivery were collected from medical record units. Data extraction tool that contains all required variables was designed and used to extract data from both the log book and the client cards. Data were collected by 8 third year resident in Gynecology and Obstetrics. Before data extraction, training was provided on data collection tool and the data collection process were conducted under the supervision of the investigators. Sample of completed data extraction tools were cross checked with original patient records and feedback was given to ensure data quality.

### Study variables

#### Independent variables

- **Socio-demographic variables**: Age, Place of Residence
- **Obstetrics/Genecology related variables**: Parity, Gravidity, Gestational Age, Having Mal-presentation, History of cesarean section scar, ANC visits, contracted pelvis, uterine anomalies, previous myomectomy
- **Management and follow up related Variables**: Oxytocin/prostaglandin misuse, uterine instrumentation, Fundal pressure application

#### Dependent variable

Uterine rupture (Yes Vs. No).

### Data analysis

The collected data were coded and entered into EPI-Info version seven then exported to STATA version 12 for cleaning and analysis. Descriptive analysis was used to explore the characteristics of mothers. The associations between uterine rupture and independent variables were modeled using binary logistic regression analysis. Bivariate logistic regression analysis was used to assess the existence of crude relationship between independent variables and uterine rupture. At this level the candidate variables for multivariate analysis were selected at *P*-value < 0.25 significance level [27]. Multivariate logistic regression was applied to estimate the adjusted effects of independent variables on uterine rupture. The association between independent variables and uterine rupture was estimated using odds ratio with 95% confidence interval. The significance of associations was declared at *p*-value of less than 0.05. The regression model was developed using backward stepwise strategy. The final fitted model was assessed for multicolliniarity using Variance Inflation Factor (VIF) [28] and goodness of fit using Hosmer and Lemishow test [29]. The model ability to correctly classify those subjects who experience outcome of interest and those who do not was assessed using Receiver Operating Characteristics (ROC) curve [28, 29]. The parsimonious model that best explain data with minimum of free parameters was selected using Akaike Information Criteria (AIC) [30].

## Results

### Socio-demographic characteristics of cases and controls

In the current study, we incorporated 144 eligible cases of uterine rupture and 288 controls with normal spontaneous deliveries. As per the results of analysis significantly higher proportion of cases of uterine rupture tended to be older and belongs to rural residential. Accordingly the study revealed that, about 74(51.39%) of uterine rupture and 92(31.9%) of controls were in the age range of 26–30 years. Among cases of uterine rupture 95(75.40%) and among controls 78(27.66%) were from a rural residence (Table 1).

**Table 1:** Socio-demographic characteristics of mothers visited Adama city public and private hospitals for delivery service during January 01, 2011 to December, 2015

Characteristics	Cases Number (%)	Controls Number (%)	Total Number (%)	Chi-Square
Maternal Age (*n* = 432)				35.44^***^
Less than 20	4 (2.78)	54 (18.75)	58 (13.43)	
21–25	28 (19.44)	88 (30.56)	116 (26.85)	
26–30	74 (51.39)	92 (31.9)	166 (38.43)	
31–35	28 (19.44)	37 (12.85)	65 (15.05)	
36 and above	10 (6.94)	17 (5.90)	27 (6.25)	
Residence (*n* = 408)				81.26^***^
Urban	31 (24.60)	204 (7.34)	235 (57.60)	
Rural	95 (75.40)	78 (27.66)	173 (42.40)	

^***^P value  < 0.001

### Obstetric characteristics of cases and controls

This study revealed that multiparous, multigravida, women with mal-presentation, women with at list one gynecologic risk factor, women with a history of CS scar and women with no history of ANC visit were highly proportionate among cases of uterine rupture compared to controls (*p*-value < 0.05) (Table 2).

**Table 2:** Obstetrics characteristics of mothers visited Adama city public and private hospitals for delivery service during January 01, 2011 to December, 2015

Characteristics	Cases Number (%)	Controls Number (%)	Total Number (%)	Chi-Square
Parity (*n* = 361)				67.23^***^
Nulliparous	3 (2.17)	71 (31.8)	74 (20.50)	
Primiparous	34 (24.64)	61 (27.35)	95 (26.32)	
Multiparous	65 (47.10)	79 (35.43)	144 (39.89)	
Grand multiparous	36 (26.09)	12 (5.38)	48 (13.30)	
Gravidity (*n* = 419)				81.08^***^
One	5 (3.65)	108 (38.30)	113 (26.97)	
2–4	79 (57.66)	146 (51.77)	225 (53.70)	
Five and above	53 (38.69)	28 (9.93)	81 (19.33)	
GA in weeks (*n* = 335)				24.74^***^
Less than 37	90 (89.11)	146 (62.39)	236 (70.45)	
37–40	5 (4.95)	55 (23.50)	60 (17.91)	
Above 40	6 (5.94)	33 (14.10)	39 (11.64)	
Mal-presentation (*n* = 409)				23.69^***^
Yes	27 (21.09)	15 (5.34)	42 (10.27)	
No	101 (78.91)	266 (94.66)	367 (89.73)	
Gynecologic factor (*n* = 265)				17.76^***^
Yes	17 (16.67)	3 (1.84)	20 (7.55)	
No	85 (83.33)	160 (98.16)	245 (92.45)	
History of CS scar (*n* = 418)				10.54^**^
Yes	14 (9.86)	7 (2.54)	21 (5.02)	
No	128 (90.14)	269 (97.46)	397 (94.98)	
History of ANC visit (*n* = 387)				59.02^***^
Yes	75 (59.52)	240 (91.95)	315 (81.40)	
No	51 (40.48)	21 (8.05)	72 (18.60)	

Gynecologic factor are: Contracted Pelvis, Uterine Anomaly, and Previous Myomectomy

^**^P value  < 0.01; ^***^P value  < 0.001

The proportion of multiparous women was 65(47.10%) among cases and 79(35.43%) among controls. About 53(38.69%) of uterine rupture cases and 28(9.93%) of controls were belonged to gravidity of five and above. The Mal - presentation was highly proportionate among cases with 27(21.09%) whereas 15(5.34%) among controls. The proportion of CS scar is higher among cases 14(9.86%) compared to controls 7(2.54%). Women with no history of ANC visit were higher in proportion among cases of uterine rupture 51 (40.48%) compared to controls 21(8.05%) (Table 2).

### Type of management performed

The study showed that, lower proportions of cases of uterine rupture 10(7.35%) were managed with oxytocin/prostaglandin compared to controls 45(16.25%). But the proportion of women managed with uterine instrumentation were higher among cases 8(5.88%) compared to controls 6(2.19%). The management of labor through fundal pressure was not much different between cases 1(1.14%) and controls 1(1.12%). The study revealed a higher proportion of cases 76 (66.09%) referred by health professionals compared to controls 117(45.17%) (Table 3).

**Table 3:** Type of managements performed for mothers visited Adama city public and private hospitals for delivery service during January 01, 2011 to December, 2015

Characteristics	Cases Number (%)	Controls Number (%)	Total Number (%)
Oxytocin use (*n* = 413)			
Yes	10 (7.35)	45 (16.25)	55 (13.32)
No	126 (92.65)	232 (83.7)	358 (86.68)
Uterine instrumentation (*n* = 410)			
Yes	8 (5.88)	6 (2.19)	14 (3.41)
No	128 (94.12)	268 (97.81)	396 (96.59)
Fundal pressure (*n* = 170)			
Yes	1 (1.14)	1 (1.12)	2 (1.18)
No	87 (98.86)	81 (98.82)	168 (98.82)
Type of management (*n* = 147)			
Total Hysterectomy	76 (53.15)	2 (50.00)	78 (53.06)
Subtotal Hysterectomy	22 (15.38)	1 (25.00)	23 (15.65)
Repair	45 (31.47)	1 (25.00)	46 (31.29)
Type of referral (*n* = 374)			
Self-referred	39 (33.91)	142 (54.83)	181 (48.40)
Referred by professionals	76 (66.09)	117 (45.17)	193 (51.60)

### Factors associated with uterine rupture

The odds of having a uterine rupture in relation to different characteristics of women were estimated by odds ratio using logistic regression analysis. Bivariate logistic regression analysis was used to select candidate variables for multivariate analysis at *p*-value less than 0.25. In the model development process the existence of multi co-linearity was assessed using VIF. The result of the assessment showed that there was strong co-linearity between parity and gravidity (mean VIF = 9.26). As a result, we exclude parity from multivariate model. The final fitted model was also tested for goodness of fit using Hosmer and Lemeshow test. The test result showed that the model became poor by the inclusion of variable called mal-presentation (*P*-value = 0.0373). Though it had statistically significant association with uterine rupture, we fitted the final model excluding mal-presentation. In the final model the odds of having a uterine rupture across each independent variable were adjusted for confounding effects. Accordingly women’s residential place, number of gravidity, history of CS scar and history of ANC visit were found to be significantly associated with the odds of having uterine rupture (*p*-value < 0.05) (Table 4).

**Table 4:** Factors associated with uterine rupture among mothers visited Adama city public and private hospitals for delivery service during January 01, 2011 to December, 2015

Characteristics	Uterine Rupture Status		COR [95%CI]	AOR [95%CI]
	Yes	No		
Maternal Age				
Less than 20	4 (2.78)	54 (18.75)	Ref.	Ref.
21–2	28 (19.44)	88 (30.56)	4.29 [1.43, 12.92]^**^	2.50 [0.38, 16.52]
26–30	74 (51.39)	92 (31.9)	10.86 [3.76, 31.36]^***^	3.67 [0.61, 22.08]
31–35	28 (19.44)	37 (12.85)	10.21 [3.31, 31.56]^***^	2.67 [0.38, 18.54]
36 and above	10 (6.94)	17 (5.90)	7.94 [2.20, 28.60]^**^	2.24 [0.25, 20.38]
Residence				
Urban	31 (24.60)	204 (7.34)	Ref.	Ref.
Rural	95 (75.40)	78 (27.66)	8.01 [4.95, 12.98]^***^	6.29 [3.39, 11.66]^***^
Gravidity				
One	5 (3.65)	108 (38.30)	Ref.	Ref.
2–4	79 (57.66)	146 (51.77)	11.69 [4.58, 29.84]^***^	8.80 [2.96, 26.12]^***^
Five and above	53 (38.69)	28 (9.93)	40.89 [14.94, 111.89]^***^	27.89 [8.42, 92.34]^***^
GA in week				
Less than 37	90 (89.11)	146 (62.39)	Ref.	Ref.
37–40	5 (4.95)	55 (23.50)	0.15 [0.57, 0.38]^***^	2.65 [0.50, 13.96]
Above 40	6 (5.94)	33 (14.10)	0.29 [0.12, 0.73]^**^	0.53 [0.07, 3.91]
Mal-presentation				
Yes	27 (21.09)	15 (5.34)	4.74 [2.42, 9.28]^***^	3.96 [1.59, 9.82]^**^
No	101 (78.91)	266 (94.66)	Ref.	Ref.
History of CS scar				
Yes	14 (9.86)	7 (2.54)	4.20 [1.65, 10.67]^**^	9.94 [3.39, 11.66]^***^
No	128 (90.14)	269 (97.46)	Ref.	Ref.
History of ANC visit				
Yes	75 (59.52)	240 (91.95)	Ref.	Ref.
No	51 (40.48)	21 (8.05)	7.77 [4.39, 13.75]^***^	9.64 [4.37, 21.29]^***^

^*^P value  < 0.05; ^**^P value  < 0.01; ^***^P value  < 0.001

As per the result of multivariate analysis being women from a rural residence were associated with 6.29 (AOR = 6.29; 95% CI: 3.39, 11.66) times higher odds of having a uterine rupture compared to urban. The odds of having a uterine rupture was 27.89 (AOR = 27.89; 95% CI: 8.42, 92.34) times higher for a woman of gravidity of five and above and 8.80 (AOR = 8.80; 95% CI: 2.96, 26.12) times higher for women of gravidity of two to four compared to primi-gravida. Having a history of cesarean section scar was associated with 9.94 (AOR = 9.94; 95% CI: 3.39, 11.66) times higher odds of having a uterine rupture compared to their counterparts. The odds of having a uterine rupture among women with history no ANC visit was 9.64 (AOR = 9.64; 95% CI: 4.37, 21.29) times higher compared to women with a history of ANC visits (Table 4).

## Discussion

The study was aimed to identify factors associated with having uterine rupture. The result of analysis showed that the likelihood of having uterine rupture was found to be associated with being women from rural residence, increase in the number of gravidity, presence of CS scar and having no history of ANC visit.

The current study revealed that the chance of having uterine rupture is higher for a woman from rural residence compared to urban, which is in line with prior studies done in Pakistan [31] and Debremarkos Ethiopia [32]. This could be due to lack of access to nearby health institution in rural residential areas. For women residing in rural area, health facilities are distant and accesses to information about institutional deliveries are limited in comparison to woman reside in urban. As a result the higher chance of uterine rupture for women from rural residents may be attributed to two delays in the process of getting obstetric cares. The first is delay to decide for seeking health care as early as possible and the second is delay in reaching health facility. Additionally, maybe there is a failure of early referral for any labor abnormality, thus resulting in delay in early intervention leading to ruptured uterus. These problems could be possibly addressed by construction of health institution nearby to the community that are capable of managing obstructed labor, increasing awareness on skilled birth attendance and establishing a referral system.

This study also showed that the chance of having uterine rupture was increased by increase in number of gravidity. Similarly, studies done in Yemen [33] and Nigeria [34, 35] revealed gravidity as one of risk factors for having uterine rupture. The abdominal wall becomes weak and lax for mothers with high number of pregnancies. As a result this contributes for the head of fetus not to be engaged early that leads to different mal-presentations. Mal-presentation was found to be one of the contributing factors for rupture in some previous studies.

In the current study uterine rupture was found to be higher among women with a history of scarred uterus. The higher chance of uterine rupture was also observed among women with history of CS scar as per the studies done in United Kingdom [1], India [36] and Nigeria [35]. Mostly woman with previous CS delivery are highly likely to develop scar dehiscence especially when the incision of uterine wall is vertical and when the inter-pregnancy interval is short after a CS scar. Then these will lead women to develop rupture.

This study also elucidated that, mothers not having ANC care were more likely to have uterine rupture. The occurrences of uterine rupture among women not having ANC patient have also been noted in other studies done in Nigeria [34, 35, 37]. The differences in the level of obstetric practices, an availability and under-utilization of the essential obstetric care services gained during pregnancy, would account for the high chance of developing uterine rupture.

## Limitation of the study

This study has some limitations. It relied on a review of logbook and medical records, in which data on some of important socio-demographic and socioeconomic information were unavailable. Additionally, data were not primarily collected for research purpose and lack completeness. Being a case control study, it is unlikely to infer a causal association and needs further study to explore the causes of having uterine rupture. With these all limitations this study will serve as baseline information for a government organization, stakeholders and decision makers working on programs targeted to minimize the odds of developing uterine rupture and its consequences.

## Conclusion

In general based on the current study, for the women living in rural residence and having larger numbers of previous pregnancies the chance to have uterine rupture were significantly higher compared to their counterparts. Similarly the chance to have uterine rupture was significantly higher for women having a history of CS scar and not using ANC. Therefore, it is mandatory to improve an access for emergency and essential obstetric care with due attention to women in rural residence. It is also important to strengthen family planning services with special attention to women of multigravida. Furthermore, strengthening antenatal care with complete packages and referral system are issues to be addressed in order to minimize the chance to have a uterine rupture.

## Abbreviations

AIC: Akaike information criterion
ANC: Antenatal care
AOR: Adjusted odds ratio
CI: Confidence interval
CS: Cesarean section
CSA: Central Statistical Agency
EDHS: Ethiopian demography and health survey
MMR: Maternal mortality ratio
OR: Odds ratio
ROC: Receiver Operating Characteristics
VIF: Variance inflation factor
